# Exploring the pathogenesis and clinical implications of asthma, chronic obstructive pulmonary disease (COPD), and asthma-COPD overlap (ACO): a narrative review

**DOI:** 10.3389/fmed.2025.1514846

**Published:** 2025-04-17

**Authors:** Kantapat Simmalee, Theerasuk Kawamatawong, Joana Vitte, Pascal Demoly, Putthapoom Lumjiaktase

**Affiliations:** ^1^Department of Pathology, Faculty of Medicine Ramathibodi Hospital, Mahidol University, Bangkok, Thailand; ^2^Division of Pulmonary and Critical Care Medicine, Department of Medicine, Faculty of Medicine Ramathibodi Hospital, Mahidol University, Bangkok, Thailand; ^3^Immunology Laboratory, University Hospital of Reims and INSERM UMR-S 1250 P3CELL, University of Reims Champagne-Ardenne, Reims, France; ^4^Division of Allergy, University Hospital of Montpellier and IDESP, University of Montpellier - Inserm, Inria, Montpellier, France

**Keywords:** asthma, asthma-COPD overlap, chronic obstructive pulmonary disease, *in vitro* models, pathogenesis, biomarkers

## Abstract

The complexity and diversity of the immune response in patients with asthma, chronic obstructive pulmonary disease (COPD), and asthma-COPD overlap present significant challenges for disease management. Relying on a limited number of biomarkers and clinical data is insufficient to fully reveal the immunopathogenesis of these diseases. However, *in vitro* technologies such as cell analysis, cytokine investigation, and nucleic acid sequencing have provided new insights into the underlying mechanisms of these diseases, leading to the discovery of several biomarkers—including cell degranulation, cell function, secreted cytokines, and single nucleotide polymorphisms—that have potential clinical implications. This paper reviews the immunopathogenesis in asthma, chronic obstructive pulmonary disease, and asthma-COPD overlap and examines the applications of recent *in vitro* models to detect candidate biomarkers that could enhance diagnostic precision, predict severity, monitor treatments, and develop new treatment strategies. A deeper understanding of the immune response in these diseases, along with the integration of *in vitro* models into clinical practice, could greatly improve the management of these respiratory diseases, making approaches more personalized and efficient.

## Introduction

Asthma and chronic obstructive pulmonary disease (COPD) are complex diseases associated with high morbidity and mortality rates worldwide. According to the Global Initiative for Asthma (GINA) 2023 (1), they serve as umbrella terms for heterogeneous characteristics that overlap in some older patients, resulting in the previously identified asthma-COPD overlap (ACO) syndrome. In 2019, the prevalence of ACO ranged from 1.4% to 2.6% in the general population; among individuals with asthma and COPD, it varied from 19.5% to 33.6% and from 19.3% to 39.9%, respectively (2). The coexistence of these two diseases leads to a specific phenotype influenced by various individual factors, including medical conditions, immune responses, and genetic variation, which contribute to worse clinical outcomes and complicate identification and treatment compared to asthma or COPD alone (3).

**Figure 1:**
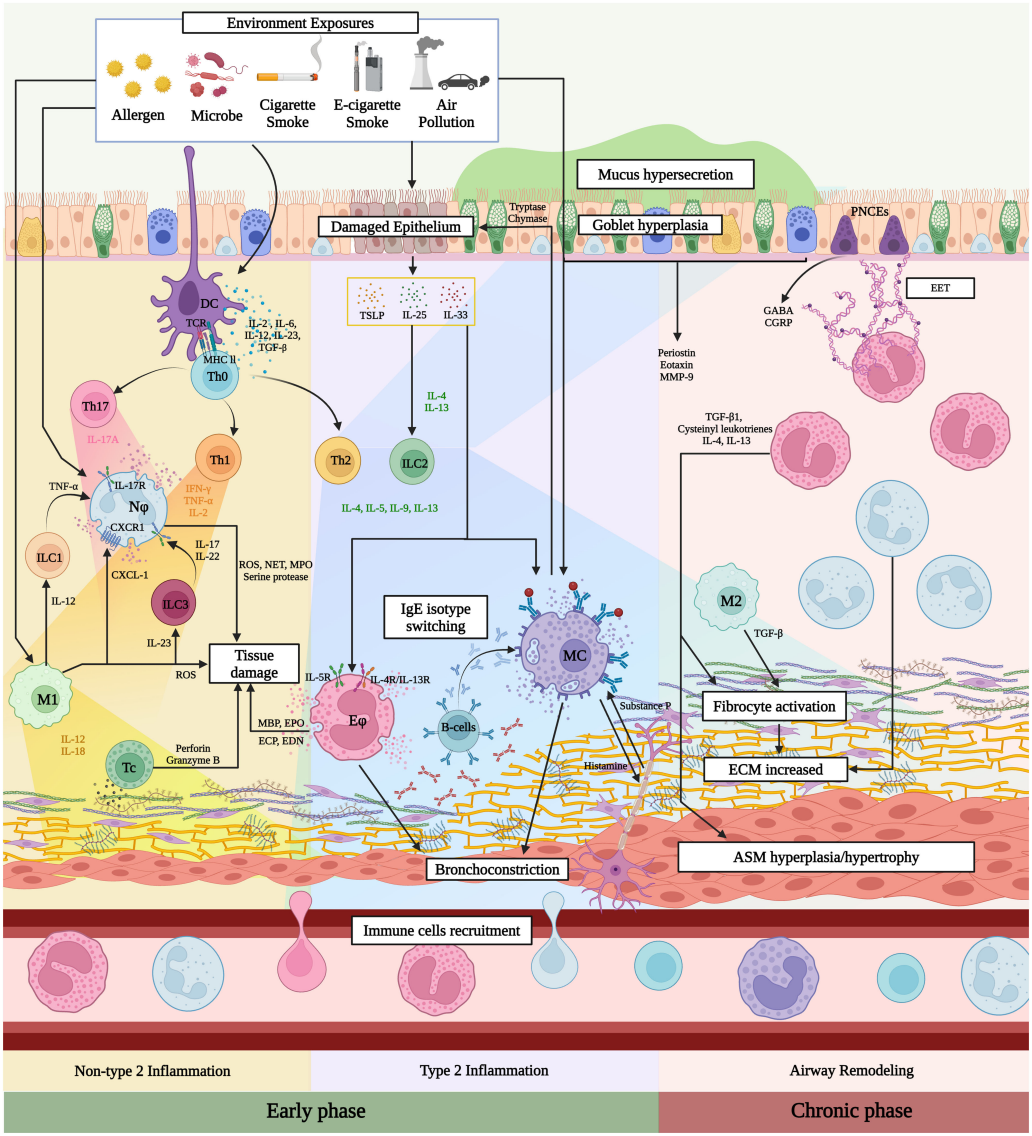
Immunopathogenesis of airway inflammation in ACO. Figure created with www.BioRender.com. Environmental exposures initiate ACO immunopathogenesis by increasing immune cell infiltration and cytokine secretion, which leads to airway inflammation. In the early stages, various cytokine chain reactions activate effector cells, resulting in tissue damage, bronchoconstriction, and mucus hypersecretion. The inflammatory signals at the damaged sites attract and activate fibroblasts, which move more slowly than immune cells. The prolonged period of chronic inflammation and repeated cycles of tissue injury and abnormal repair processes induce airway remodeling by increasing the extracellular matrix (ECM), airway smooth muscle (ASM) hyperplasia/hypertrophy, and goblet cell hyperplasia, ultimately causing permanent obstruction. DC, Dendritic cell; TCR, T-cell receptor; TSLP, Thymic stromal lymphopoietin; EET, Extracellular matrix; GABA, Gamma-aminobutyric acid; CGRP, Calcitonin gene-related peptide; Th1, T-helper cell type 1; Th2, T-helper cell type 2; Th17, T-helper cell type 17; Treg, regulatory T-cell; Tc, Cytotoxic T-cell; ILC1, Innate lymphoid cell type 1; ILC2, Innate lymphoid cell type 2; ILC3, Innate lymphoid cell type 3; Nϕ, Neutrophil; MC, Mast cell; M1, Alveolar macrophage type 1; M2, Alveolar macrophage type 2 ECM, extracellular matrix; ASM, airway smooth muscle.

The majority of ACO cases often present airway obstruction and respiratory symptoms due to inflammation from excessive immune responses, which are linked to worse outcomes (4, 5). Environmental exposures to microbes, allergens, and air pollution—including cigarette smoke, electronic cigarettes, and particulate matter with a diameter of 2.5 μm or smaller (PM_2.5_)—induce cooperation between innate and adaptive immune responses, leading to persistent airway inflammation and remodeling by recruiting immune cells and releasing cytokines that differ in inflammatory characteristics (6, 7) (Table 1, Figure 1).

**Table 1:** Role and characteristics of inflammatory players in asthma, COPD, and ACO.

**Immune response**		**Disease characteristics**			**Dysfunction's effect on immunopathogenesis**	**References**
		**Asthma**	**COPD**	**ACO**		
**Innate immune cells**						
Eosinophil		++++	+	++++	•Correlate with exacerbation frequency •Airway hyperresponsive •Increase type-2 cytokine production •Tissue damage •Mucus hypersecretion •Airway remodeling	(10, 15, 163–166)
Neutrophil		++	++++	++++	•Tissue damage and remodeling •Associate with emphysema •Cytotoxic activity	(46, 164, 165)
Mast cell		+++	+++	++	•Correlation with the allergy phenotype •Increase airway hyperresponsiveness and type-2 cytokines •Induce airway epithelial barrier disruption •Contribute to fibrosis and airway remodeling via fibroblast crosstalk and epithelial migration	(9, 167–171)
ILC1		++	+++	N/A	•Increase neutrophil activation via Th1 cytokines	(59, 67, 68, 172, 173)
ILC2		++++	+	N/A	•Increase type 2 inflammation via Th2 cytokines	(68, 172, 173)
ILC3		++	++	N/A	•Increase neutrophil activation via Th17 cytokines	(59, 67, 73, 172)
Macrophage		+++	++	++++	•Tissue damage •Fibroblast activation leading to airway remodeling •Th1 and Th2 polarization	(9, 174)
Dendritic cell	cDC1	++	+++	N/A	•Initiate T-cell activation via MHC molecule and co-stimulating cytokines	(175–179)
	cDC2	+	++	N/A		
	moDC	N/A	N/A	N/A		
	pDC	+	++	N/A		
**Adaptive immunity**						
Th1		++	+++	N/A	•Drive non-type 2 inflammation phenotype and cell recruitment via pro-inflammatory cytokines and chemokines	(81, 83, 85, 180, 181)
Th2		+++	+	N/A	•Drive type-2 inflammation phenotype and cell recruitment via pro-inflammatory cytokines and chemokines	(81, 83, 85, 172, 180)
Th17		++	+++	N/A	•Drive non-type 2 inflammation phenotype and cell recruitment via pro-inflammatory cytokines and chemokines	(81, 83, 180–183)
Treg		++	+	N/A	•Reduce immune suppressive function, leading to immune overactivation	(83, 180, 182, 183)
Tc		+	++++	++	•Increase cytotoxic activity, causing tissue damage and emphysema	(9, 183–185)
B-cell		++	+	N/A	•Increase IgE secretion associated with airway hyperresponsiveness •Autoantibody production	(185, 186)
**Cytokines**						
IL-4		+++	+	++++	•Th2 polarization •Airway hyper-responsiveness •Tissue remodeling •IgE and IgG1 switching •Mast cell proliferation	(163, 187)
IL-5		+++	+	++	•Eosinophils proliferation, maturation, survival, and activation	(10, 87, 187, 188)
IL-13		++++	+	++	•Th2 polarization •Airway hyper-responsiveness •Tissue remodeling •Goblet cell hyperplasia •ASM hypertrophy and hyperplasia	(87)
IL-8 (CXCL-8)		+++	++++	++++	•Neutrophil recruitment	(87, 163)
IL-17		+	++++	++	•Fibrocyte proliferation •Neutrophil recruitment and activation •ILC3 polarization	(87, 189)
IL-6		++++	++	+++	•Neutrophil activation	(10, 87, 188)
TNF-α		++++	++	+++	•Neutrophil activation •Macrophage activation	(87, 163, 189)
IFN-γ		+	+++	++	•Neutrophil activation	(187)
TGF-β		++	++++	+++	•Airway remodeling •Treg polarization •M2c differentiation	(10, 187)
**Immune mediators**						
Periostin		++++	++	+++	•Mucus hypersecretion •goblet cell hyperplasia •eosinophil recruitment •Airway remodeling	(15, 87)
YKL-40		++	++++	+++	•Airway remodeling •Airway inflammation	(10, 15, 166)
Nitric oxide		++++	++	++	•Type 2 inflammation •Vasodilation •Bronchodilator	(87, 190)

N/A, not applicable; + to ++++, from suggested to the proven role compared between asthma, COPD, and ACO.

This article focuses on novel data regarding immunopathogenesis, laboratory examination, and biomarkers in ACO patients based on *in vitro* models. Although techniques such as flow cytometry, enzyme-linked immunosorbent assay (ELISA), cytology, histology, and molecular methods are well established in clinical research for understanding ACO diversity, they also shed light on their potential for future clinical applications (8–10). A better understanding of ACO immunopathogenesis through these models aims to establish more effective stratification criteria and identify accurate biomarkers, ultimately improving diagnosis, prediction, prognosis, monitoring, and the identification of novel treatment targets.

## The innate immune response in asthma, COPD, and ACO

### Airway epithelial cells (AECs)

AECs respond to environmental exposures by releasing alarmins, including thymic stromal lymphopoietin (TSLP), IL-25, and IL-33, which activate type 2 innate lymphoid cells (ILC2s), eosinophils, and mast cells (MCs), driving inflammation and immune cell recruitment (11). In asthma-chronic obstructive pulmonary disease overlap, AECs increase the secretion of other Damage-Associated Molecular Patterns (DAMPs), such as high mobility group box 1 protein (HMGB1), 70-kilodalton heat shock proteins (HSP70), cathelicidin LL-37 (LL-37), and calcium-binding protein A8 (A8), leading to the recruitment of effector cells and the initiation of inflammation (12). Additionally, epithelial cells and other cell types, such as basal, club, ciliated, goblet, ionocytes, endothelial, and fibroblast cells, are induced by IL-4, IL-13, and TGF-β to secrete periostin. The overexpression of periostin results in mucus hypersecretion, goblet cell hyperplasia, and increased matrix metalloproteinase (MMP)-9 secretion, contributing to eosinophil inflammation and permanent airway obstruction (13). IL-4 and IL-13 also trigger AECs to produce eotaxin, which attracts fibroblasts and eosinophils to the inflamed site (14). Moreover, activated AECs, neutrophils, and macrophages can secrete YKL-40, correlating with airway inflammation and remodeling (15).

Cilia on AECs are vital in respiratory disease. Motile cilia play a critical role in lung homeostasis by beating the mucus layer, which traps inhaled pathogens, toxins, and particles out of the airway. Abnormalities in ciliary function, quantity, and structure impair mucus clearance, increasing susceptibility to infections and promoting chronic inflammation (16, 17). Primary cilia act as cellular antennas, influencing the differentiation, migration, and cell fate control of AECs, airway smooth muscle (ASM) cells, and fibroblasts (18). Interestingly, although the quantity of primary cilia does not differ in normal epithelium, it significantly increases in COPD, specifically in the remodeling area (18). The increased antennas may contribute to dysregulated tissue repair, leading to abnormal differentiation and proliferation, ultimately resulting in airway thickening.

In the alveoli, alveolar type 1 cells (AT1) facilitate gas exchange, while alveolar type 2 cells (AT2) repair tissue damage through self-renewal, differentiation into AT1, secretion of TGF-β, and production of surfactant. Cigarette smoke and PM_2.5_ have been shown to induce apoptosis in AECs and disrupt the differentiation of AT2 into AT1, leading to pulmonary emphysema in COPD murine models (19).

### Dendritic cells (DCs)

DCs identify ligands through pattern recognition receptors (PRRs), which trigger them to co-activate naïve helper T cells (Th0) via antigen presentation and cytokine secretion. The cytokines produced by DCs depend on the types of antigens and the subpopulations of DCs, creating the microenvironment around the site of inflammation (20). cDCs can be classified into conventional DCs type 1 (cDC1), conventional DCs type 2 (cDC2), plasmacytoid DCs (pDCs), and monocyte-derived DCs (moDCs) (21). cDC1s and moDCs are the primary sources of IL-12, IFN-α, and IFN-β, which activate T helper type 1 (Th1) polarization. Additionally, they cross-present antigens to CD8^+^ cytotoxic T cells (Tc), leading to tissue damage in the lungs (20, 21). In contrast, studies in asthmatic mice have shown that cDC2s and moDCs play significant roles in T helper type 2 (Th2) polarization (22). cDC2s also contribute to promoting T helper type 17 (Th17) polarization in lung asthmatic mouse models (23). pDCs produce type-I IFN, TNF-α, and IL-6, inducing Th1, Th17, and regulatory T cell (Treg) polarization (24).

Dysfunction of DCs contributes to the progression of asthma, COPD, and ACO. An imbalance in DC subpopulations correlates with COPD severity, as indicated by the ratio of OX40L to programmed death-ligand 1 (PD-L1) (25). Moreover, reduced DC activation leads to a decrease in Th2 and Th17 signaling, as demonstrated by a reduction in mucus production and cellular recruitment (26).

### Alveolar macrophages (AMs)

AMs capture and eliminate foreign antigens while also repairing damaged tissue. Upon activation, they polarize into two subtypes: classically activated macrophages (M1) and alternatively activated macrophages (M2) (27). M1 is triggered by lipopolysaccharide (LPS) and cytokines such as TNF-α, IFN-γ, and IL-12. This subclass can eliminate bacteria, damaged cells, and tumors, and it plays a role in Th1 activation through the secretion of IL-1β, IL-6, IL-12, IL-18, IL-23, TNF-α, inducible nitric oxide synthase (iNOS), reactive oxygen species (ROS), CXCL-1, CXCL-2, CXCL-3, CXCL-9, and CXCL-10 (27–30). Conversely, M2 is notable for its role in tissue repair and maintaining immune homeostasis through the secretion of IL-10, TGF-β, and vascular endothelial growth factor (VEGF) (29, 30). M2 can be divided into four main subgroups: M2a, M2b, M2c, and M2d. M2a polarization, influenced by IL-4 and IL-13, functions in tissue repair by secreting TGF-β, insulin-like growth factor, and fibronectin. M2b is induced by immune complexes, toll-like receptor (TLR) ligands, or IL-1R ligands and regulates the immune response through IL-10. M2c requires IL-10 and TGF-β to differentiate, producing the anti-inflammatory cytokine IL-10 and the tissue remodeling cytokine TGF-β. M2d, induced by IL-6, TLR ligands, and the A2 adenosine receptor (A2R), produces IL-10, TGF-β, monocyte colony-stimulating factor (M-CSF), and VEGF (31–33).

At a steady state of lung tissue, the major population of AMs exhibits a hybrid of M1 and M2 surface markers, allowing them to polarize swiftly (34). However, dysregulation in M1/M2 polarization is implicated in asthma, COPD, and ACO. Studies of small airway tissue and bronchoalveolar lavage (BAL) samples from smokers and COPD populations indicate that M1 predominates in the airway wall, while M2 phenotypes and their associated cytokines are elevated in the luminal space compared to normal controls. This suggests that the overexpression of M2 switching in the lumen produces excessive tissue repair following M1-mediated tissue damage, contributing to lung fibrosis and permanent obstruction (35).

### Eosinophils

Eosinophils respond to IL-3, IL-5, and granulocyte-macrophage colony-stimulating factor (GM-CSF), all of which are crucial for their proliferation, maturation, survival, and activation. They are also activated by TSLP and IL-33. This activation leads to inflammation, tissue injury, and airway remodeling through their adhesion, degranulation, cytokine secretion, and chemotaxis involving CCL-1, CCL-5, CCL-7, and CCL-8 (36–39). Eosinophil granules contain proteins, including major basic protein (MBP), eosinophil cation protein (ECP), eosinophil peroxidase (EPO), and eosinophil-derived neurotoxin (EDN), which affect the immunopathogenesis of ACO through their cytotoxic functions and induce inflammation (38, 40, 41). Moreover, recent reports indicate that cadherin L, derived from eosinophils, also promotes emphysema in COPD with eosinophilia (42).

Eosinophil-derived mediators, such as TGF-β1, cysteinyl leukotrienes, IL-4, and IL-13, drive the proliferation of ASM and fibroblasts, leading to long-term structural changes (38). Additionally, eosinophils interact with the neuroimmune system by forming eosinophil extracellular traps (EETs), which activate pulmonary neuroendocrine cells (PNCEs) to secrete calcitonin gene-related peptide (CGRP) and γ-aminobutyric acid (GABA), promoting cell infiltration and cytokine release, thereby increasing inflammation and mucus production (4). These non-selective mechanisms of eosinophil granule toxicity cause tissue damage and hyperresponsiveness, resulting in an increased exacerbation rate of ACO (40, 41, 43).

### Neutrophils

Neutrophils are stimulated by extracellular matrix (ECM) proteins, cytokines, and microorganisms at inflammatory sites. Optimal activation requires a two-step process involving priming and activating stimuli. Several factors, such as TNF-α, GM-CSF, IFN-γ, IL-6, IL-17, CXCL-1, and CXCL-8, have been identified as priming agents that facilitate full activation alongside DAMPs and pathogen-associated molecular patterns (PAMPs) (44, 45). Activated neutrophils release serine proteases, including neutrophil elastase (NE), cathepsin G (CG), proteinase 3 (PR3), reactive oxygen species (ROS), myeloperoxidase (MPO), and neutrophil extracellular traps (NETs). These proteases, along with ROS formation and NETosis, degrade elastin, collagen, fibronectin, and proteoglycans, contributing to epithelial cell destruction and structural changes (46). Moreover, neutrophils can initiate tissue repair through heat shock signaling, controlling the movement of the matrix from healthy areas into injured tissue (47).

Recent research shows that ECM proteins influence neutrophil functions, migration, ROS production, MPO secretion, and NET formation. Type III collagen in lung tissue reduces neutrophil migration while enhancing ROS production, leading to tissue damage and prolonged neutrophil presence in the lung tissue, contributing to long-term inflammation and airway remodeling. This may relate to the increased production of type I and type III collagen in the airway during the early stages of COPD (48). ECM also determines the velocity and direction of neutrophil migration, guiding them to the injury site and causing them to remain in the tissue (49). These neutrophil functions may explain the severity of ACO patients due to increased neutrophil attraction and activation at the inflamed site.

### Mast cells (MCs)

MC activation occurs due to various stimuli, including IL-3, IL-9, IL-33, IgE, and DAMPs (50). Once activated, they produce histamine, tryptase, chymase, leukotrienes C4, and prostaglandins D2, IL-4, IL-5, IL-13, VEGF-A, VEGF-C, and CXCL-1. These molecules are associated with inflammation and airway remodeling (50–52). Interestingly, MC-released mediators can crosstalk with neurons, inducing them to release the neuropeptide substance P, thereby triggering MC degranulation (51).

MCs have two main subpopulations defined by their content granules of tryptase and chymase: MC_T_ (tryptase-positive) and MC_TC_ (both tryptase and chymase-positive). In asthma and COPD, MC_TC_ populations are increased, affecting bronchial epithelial cells by altering their migration, velocity, proliferation, and morphology, thereby disrupting the epithelial barrier through the production of tryptase and chymase (53). The granules of MCs play a significant role in airway hyperresponsiveness, inflammation, and airway thickening. Histamine induces ASM contraction, while leukotrienes and prostaglandins mediate bronchoconstriction and remodeling (54, 55).

### Innate lymphoid cells (ILCs)

ILCs are innate immune cells that differentiate into five subsets: ILC1, ILC2, ILC3, NK cells, and lymphoid tissue inducer (LTi) cells. They express cytokines similar to Th1, Th2, and Th17 but are distinguished from T cells by their lack of specific antigen receptors, especially T cell receptors (TCRs) (56). Although the mechanisms of ILCs in ACO are still being explored, their cytokines can induce several cells to function, such as neutrophils, eosinophils, MCs, AMs, T cells, and B cells, leading to airway inflammation and remodeling in asthma and COPD. ILC1 and NK cells share the expression of IFN-γ and TNF-α via T-bet driven by IL-12 (52, 57). The cytokines released by ILC1s, such as IFN-γ and TNF-α, increase neutrophilic inflammation (58, 59). NK cells contribute to tissue damage by releasing perforin and granzyme A and B, which correlates with the risk of COPD exacerbation (60). ILC2s release IL-4, IL-5, IL-9, and IL-13 through GATA-3 activation when exposed to TSLP, IL-25, and IL-33 (56, 61). ILC2 and type 2 cytokines are increased in the peripheral blood of COPD patients (62). The increased eosinophil count correlates with ILC2 levels in asthmatic patients (63). Moreover, ILC2 also plays a role in epithelial barrier destruction, inducing inflammation in murine asthma lung tissue (64). ILC3 and LTi secrete IL-17 and IL-22 through ROR-γt activation when stimulated with IL-2 and IL-23, contributing to neutrophilic inflammation and recruitment (52, 65, 66). A positive correlation has been observed between the ILC3 population, neutrophils, and AM1 in severe asthma (67).

These ILC subtypes are not permanent and can switch to other subtypes (68). Research has found that ILCs increasingly transition into the ILC1 subclass, with the elevated ILC1 population associated with smoking status and disease severity in COPD (59). Interestingly, ILC2s can polarize into the ILC1 subtype in response to infections or exposure to toxic agents in COPD (68). This explains why individuals with the same phenotype exhibit different characteristics.

In asthma, the elevated number of ILC2s and the overexpression of type 2 cytokines correlate with greater disease severity and progression (63, 68–71). In contrast, the levels of ILC1s and ILC3s cytokines are elevated in ACO and COPD patients (72). Moreover, an increased ILC3 population and neutrophil inflammation are observed in the neutrophil asthma and COPD groups (66, 73). These changes are affected by the overexpression of specific cytokines, which can excessively drive ILC plasticity into subgroups under different conditions.

### Roles of adaptive immunity in asthma, COPD, and ACO

#### T-cells

Helper T-cells (Th) can polarize into subsets such as Th1, Th2, Th17, and Treg. Th1 cells, activated by IL-12 through STAT-4, express IFN-γ, TNF-α, and IL-2, inducing neutrophil inflammation (74). Th2 cells express IL-4, IL-5, IL-9, and IL-13 through the activation of GATA-3 upon exposure to IL-4. Th2 cytokines play an important role in disease pathophysiology by regulating eosinophil proliferation, maturation, survival, and activation through IL-5. IL-4 and IL-13 induce airway hyper-responsiveness (AHR), attracting eosinophils and neutrophils, resulting in bronchial contraction, increased bronchial ECM, and the secretion of TGF-β1 from fibrocytes (45, 75, 76). Th17 differentiation, driven by IL-6, IL-21, IL-23, and TGF-β, results in the secretion of IL-17A, IL-21, and IL-22 through STAT-3 activation. This process induces fibrocytes to release TNF-α, CXCL-1, and CXCL-8, contributing to neutrophil inflammation (45, 77). Treg cells, activated by IL-2, IL-10, and TGF-β, prevent excessive tissue damage by releasing important cytokines, such as IL-10, IL-35, and TGF-β, to inhibit T-cell polarization and promote Treg differentiation (77, 78). Reduced IL-10 levels are found in patients with poor lung function (79). Tc cells produce IFN-γ, TNF-α, perforin, and granzyme B after receiving antigens on MHC class I and co-stimulation with IL-12, IL-15, and IL-18, inducing epithelial cell apoptosis and contributing to emphysema (80).

In respiratory diseases, T-cell imbalance affects the pathogenesis of asthma and COPD (81–85). COPD patients exhibit elevated Th1 and IFN-γ levels, which are associated with decreased lung function (85). Another study involving asthma patients shows that a high level of Th17 correlates with COPD and asthma severity by promoting neutrophil infiltration, thus contributing to airway remodeling (83, 86). Moreover, COPD patients with high Th1 and low Th17 experience increased severity and frequency of exacerbations compared to those with high levels. These patients have inadequate Th1 and Th17 responses to combat infections (82). This may explain why some patients with a Th1 phenotype have frequent exacerbations due to infections. In addition, asthmatic patients demonstrate an imbalance with an elevated Th2/Th1 ratio, highlighting the Th2 role in eosinophil inflammation and airway remodeling (84, 87, 88).

Moreover, the absence of regulatory mechanisms for Treg maintains inflammation in the long run. COPD patients with rapid declines in lung function exhibit low levels of Treg (89). The decrease in the function of Treg leads to dysregulation in the immune response, highlighting the need for future studies on Treg function and personalized approaches for asthma, COPD, and ACO (87, 90).

#### B-cells

B cells produce antibodies against antigens, activated via surface antigen receptors and signals from Th cells and CD40. Once activated, B cells proliferate and retain high-affinity B-cell receptors as memory B cells residing in tissue. In lung tissue, resident B cells are stimulated by local antigens and release immunoglobulins such as IgE, which are sensitized on mast cells for degranulation (91). Moreover, B cells may contribute to airway inflammation through IgA secretion after receiving signals from epithelial cells, including IL-6, B-cell activating factor (BAFF), and a proliferation-inducing ligand (APRIL) (92, 93). An increase in IgA^+^ memory B cells correlates with decreased lung function in severe asthma (94). Despite their role in disease progression, recent data highlight their regulatory function in inflammation and airway remodeling processes, suggesting potential therapeutic uses in managing diseases (95, 96).

## Novel tools for assessing immunopathogenesis in asthma, COPD, and ACO

Current guidelines from GINA and the Global Initiative for Chronic Obstructive Lung Disease (GOLD) primarily rely on clinical data for managing asthma, COPD, and ACO, including spirometry and a few biomarkers such as blood and sputum eosinophils, IgE, and the fractional exhaled nitric oxide test (FeNO). These limited details prove insufficient for effective disease management and highlight the immune diversity present in asthma, COPD, and ACO (97). Recent *in vitro* tools provide comprehensive information that enhances disease management through a better understanding of immunopathophysiology, enabling clinicians to implement more precise and personalized treatment strategies based on potential biomarkers for diagnosis, monitoring, prognosis, and treatment.

### Epithelial function study

#### Air–liquid interface (ALI) cell culture

The ALI model mimics the lung environment *in vitro*, enabling cells to differentiate and form a functional epithelial layer. This model allows researchers to directly investigate the effects of drugs and substances on epithelial cells without dilution from culture media. Moreover, the ALI model provides insights into the hydration status of the mucus layer under confocal microscopy (98).

#### High-speed digital video microscopy

High-speed digital video microscopy analyzes the functions of motile cilia by recording video and playing it back in slow motion to detect abnormalities in cilia beating. This technique allows us to quantify cilia beat frequency (CBF) and evaluate the abnormality of the cilia beat pattern as a percentage of dyskinetic cilia, referred to as the dyskinetic index (DI). Additionally, the population of immotile cilia is measured and reported as a percentage, known as the immotile index (16).

#### Transmitted electron microscopy (TEM)

TEM can investigate abnormalities in the structure of cilia and epithelial cells, including defects in ciliary axonemes such as microtubule and dynein arm defects. It can assess disruptions in epithelial integrity by evaluating cellular extrusion and cytoplasmic blebbing and visualize damage to the mitochondria, which serve as the energy source for cilia to function normally (16).

### Enzyme-linked immunosorbent assay (ELISA)

ELISA is a technique used to monitor inflammation in respiratory diseases by quantifying substances such as proteins and cytokines. For example, sputum periostin indicates fixed airflow obstruction and correlates with elevated levels of sputum IL-13 and eosinophils (13, 99). ELISA can also detect soluble cytokine receptors, such as sIL-2R levels, which are linked to disease severity in the COPD group (100). This method also measures the levels of EETs and NETs (4). Additionally, the ELISA method can detect specific IgE (sIgE), which is essential for allergy evaluation (101).

### Flow cytometry

Flow cytometry is a technology used to analyze and classify cells based on their expressions, such as DNA/RNA content, CD markers, transcription factors, cytokines, and cell receptors. This provides more information about cell state, cell subtype, cell function, and cytokine production (4, 9).

**Figure 2:**
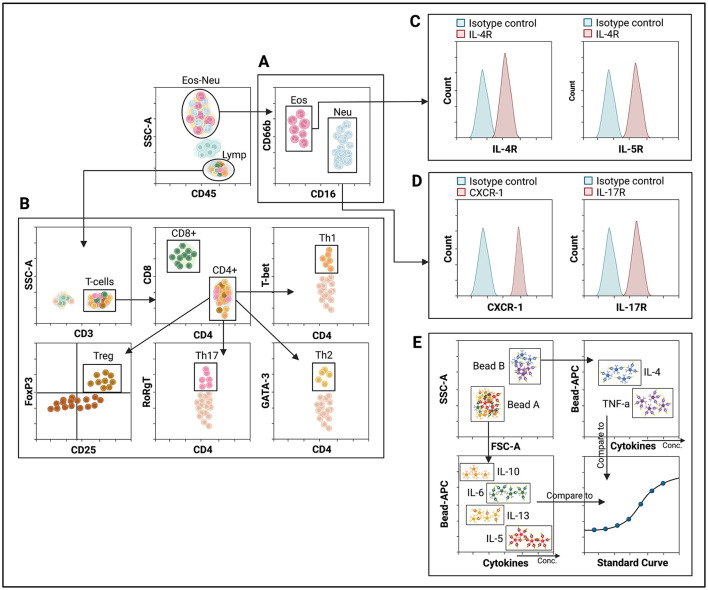
Flow cytometry data demonstrating clinical utility in asthma, COPD, and ACO. **(A)** Identification of eosinophil and neutrophil populations based on surface protein expression. **(B)** Classification of T-cell subsets based on both surface and intracellular protein expression. **(C)** Analysis of eosinophil receptor expression; higher fluorescent intensity indicates the percentage of positive cells **(D)**. In the analysis of neutrophil receptor expression, increased fluorescence intensity signifies a higher percentage of receptor-positive cells. **(E)** Cytokine analysis using bead-based assays; beads A and B are distinguished by size. Each detector bead has a unique fluorescence intensity (Bead-APC), and cytokine concentrations are quantified based on the fluorescence intensity of the secondary antibody. Eos, Eosinophils; Neu, Neutrophils.

Flow cytometry effectively quantifies the total number of specific cell types by highlighting their distinguishing features (34) (Figures 2A, B). It also reveals the percentages of surface receptors present on immune cells (Figures 2C, D). Markers such as IL-4R, IL-5R, CXCR1, and IL-17R are essential in severe obstructive lung disease, serving as targets for biological therapy that can potentially influence patients' dose response (102).

Furthermore, flow cytometry detects biomarkers using multiplex bead-based indirect immunofluorescence assays, determining biomarker levels based on fluorescent intensity on cytokine-specific beads (Figure 2E) (103). It also tracks cell proliferation, such as in the lymphocyte transformation test (LTT), which uses carboxyfluorescein diacetate succinimidyl ester (CFSE) dye to monitor lymphocyte generation. Flow cytometry also provides cell function analysis, such as the MCs activation test (MAT), which can improve the diagnosis of IgE-mediated allergic phenotypes and analyze EETs and NETs formation in specific cell populations (4, 104).

### Immune cell function tests

#### Adhesion assay

An adhesion assay evaluates how effectively stimulated cells, such as eosinophils and neutrophils, attach to the airway structure using their surface receptors. The results are reported as the percentage of adherent cells detected through their cell functions or total cell viability from all added cells. The increase in eosinophil and neutrophil adhesion correlates with higher eosinophil and neutrophil counts and infiltration into inflamed lung structures in obstructive lung diseases, which leads to airway remodeling in persistent inflammation (38).

#### Degranulation assay

Currently, several methods for investigating degranulation activity are available, such as flow cytometry and ELISA. Flow cytometry techniques detect molecule production during degranulation, noting surface markers (e.g., CD63, CD107a, and CD203c) and intracellular changes (e.g., histamine and calcium concentration) (105). Additionally, the ELISA method can be used to directly detect degranulation-specific proteins, such as ECP, MPO, and tryptase (105, 106).

#### Chemotaxis assay

A chemotaxis assay analyzes cell direction, migration, and velocity in response to a chemoattractant. The dysfunction of cell migration leads to severe chronic inflammation and increases airway remodeling in obstructive lung disease (107). An outstanding feature in recent years is microfluidic-based migration assays, which can simulate the 3D microenvironments of human lung tissue. Unlike the 2D system that only enables cell adhesion and migration on surfaces, the 3D design mimics lung tissue conditions by allowing cells to interact with an ECM-like matrix. Cell migration is recorded through live-cell videos and images to measure migration percentage, total distance, direction, speed, and velocity (49).

### Molecular analysis

Molecular technologies, such as nucleic acid sequencing and real-time polymerase chain reaction (RT-PCR), shed light on how single nucleotide polymorphisms (SNPs) play a role in immune system dysregulation in various diseases. SNPs are variations in a single nucleotide within human DNA that can lead to differences in individual phenotypes. Numerous studies have demonstrated the significance of SNPs in assessing disease risk and severity, as well as in informing treatments for asthma, COPD, and ACO, which are critical for future clinical practice (108). For example, gene variations associated with the IL-4/IL-13 pathway are linked to asthma susceptibility. Chinese patients with four SNPs, including *IL4* (rs2243250C>T), *IL13* (rs1800925C>T), *IL4R* (rs1805010G>A), and *STAT6* (rs3224011T>C), show an association with a high-risk genotype for asthma vulnerability by elevating the expression levels of *IL4, IL13*, and *STAT6* (108). Variations in *TNF* (rs1800629G>A) are associated with an increased risk of COPD in Asian populations (109). The haplotype of *VEGFA* (rs833068G>A, rs833070T>C, rs3024994C>T, rs3024997G>A, and rs3025000C>T), designated as GCCAT, significantly increases COPD risk in the Mongolian population, with an odds ratio (OR) of 3.409 (110). Furthermore, research on type-2 phenotype asthma and COPD among the Japanese population indicates that *IL4R* (rs8832A > G), associated with IL-4Rα levels and frequent exacerbations, may predict the effectiveness of IL-4Rα antagonist drugs (111).

## Clinical implications of asthma, COPD, and ACO immunopathogenesis findings

To date, the clinical information obtained from routine medical practice remains insufficient for effectively managing and improving the clinical outcomes of asthma, COPD, and ACO patients. However, the majority of clinical treatment recommendations and practice guidelines depend on the phenotypes rather than the endotypes of asthma, COPD, and ACO (99).

**Figure 3:**
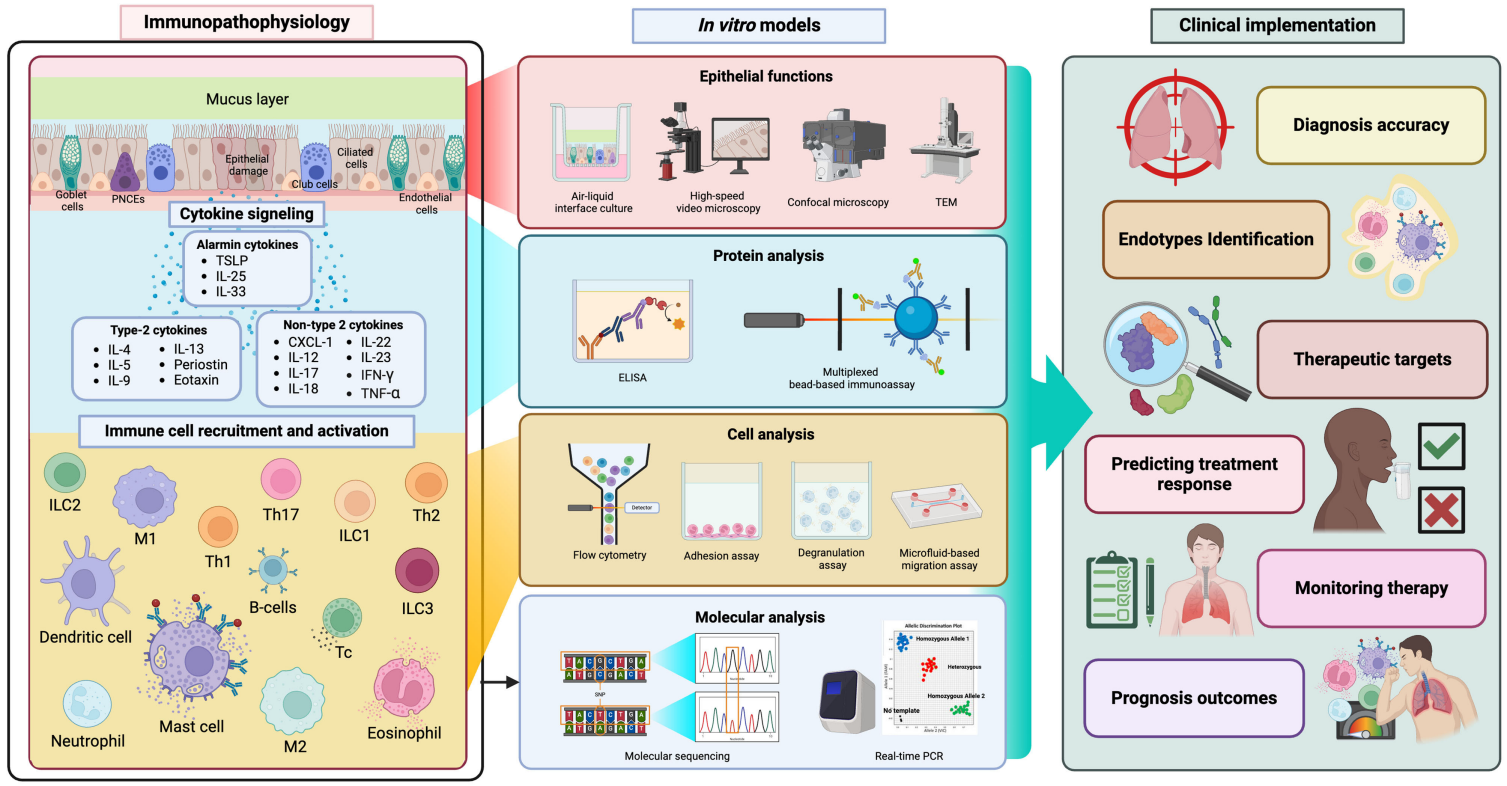
*In vitro* models bridging immunopathogenesis insights to clinical practice. Figure created with www.BioRender.com. *In vitro* models translate complex immune pathophysiology into clinical practice for asthma, COPD, and ACO. These models improve diagnostic accuracy, endotype identification, therapeutic target discovery, treatment-response prediction, intensive monitoring, and precise prognostic outcomes.

Laboratory investigations, including biomarkers that represent the immunopathogenesis of asthma, COPD, and ACO, are sophisticated tools used to reveal the underlying individual characteristics in clinical practice. These phenotypic approaches and biomarkers, such as eosinophil count, sIgE, and FeNO, are widely integrated into routine clinical practice (112). The purpose of these biomarkers is to diagnose and classify obstructive airway diseases, such as type-2 inflammatory patterns that include allergic and eosinophilic phenotypes among type-2 high asthmatic patients (113). Moreover, they help predict the outcomes or prognosis of the diseases. Elevated levels of type-2 biomarkers, despite standard care in severe asthma, are associated with poor clinical outcomes (114). More importantly, biomarkers are also utilized to monitor the magnitude of underlying systemic and local disease activity (115). Ultimately, their clinical applicability lies in predicting treatment response with disease-specific treatment modalities (116, 117). The immune response is a critical determinant influencing disease outcomes. Given the current insights into immunopathogenesis, there is a growing recognition of the vital role that *in vitro* models play in enhancing clinical practice. Therefore, *in vitro* tools are emerging to fill the gap in our understanding of these complexities and clarify clinical management by identifying and targeting individual characteristics (118) (Table 2, Figure 3).

**Table 2:** Utility of example biomarkers in asthma, COPD, and ACO.

**Clinical application**	**Biomarkers/Targets**		**Phenotypes**	**Role/potential use**
Diagnosis	IL-8 (CXCL-8)		Non-type 2	Show diagnostic performance in the ACO group with the area under the curve (AUC) of 0.68 (164).
	NAGL		Non-type 2	Identifies ACO from asthma with an AUC of 0.75 (166).
	*TNF*	rs1800629G>A	Non-type 2	Higher risk genotype for COPD susceptibility in Asian populations. The A allele is associated with a 2.45-fold increased risk of COPD compared to the G allele in a meta-analysis (109, 191).
	Periostin		Type 2	Surrogate marker for type-2 high phenotypes (15, 99). Show diagnostic performance in asthma with an AUC of 0.87 (192).
	*IL13*	rs1800925C>T	Type 2	Higher risk genotype for asthma susceptibility from higher expression levels of *IL4, IL13*, and *STAT6* (108).
	*IL4R*	rs1805010G>A		
	*IL4*	rs2243250C>T		
	*STAT6*	rs3224011T>C		
	YKL-40		Type 2	Identifies ACO from COPD with an AUC of 0.71 (166).
	*VEGFA*	rs833068G>A	Type 2/Non-type 2	The haplotype of GCCAT significantly increases COPD risk with an odds ratio (OR) of 3.409 in the Mongolian population (110).
	*VEGFA*	rs833070T>C		
	*VEGFA*	rs3024994C>T		
	*VEGFA*	rs3024997G>A		
	*VEGFA*	rs3025000C>T		
	VEGF-A		Type 2/Non-type 2	Show diagnostic performance in the ACO group with an AUC of 0.65 (164).
Prognosis	ILC1 and ILC3		Non-type 2	Prognosis severity linked to cigarette smoke (59, 67).
	TGF-β:IL-35		Non-type 2	Prognosis marker for fibrosis development (78).
	*IL4R*	rs8832A>G	Type 2	Correlated with frequent exacerbations (111).
	Eosinophil adhesion		Type 2	Marker for exacerbation and airway remodeling (38).
	Periostin		Type 2	Marker for airway remodeling prediction (15).
	IL-10		Type 2/Non-type 2	Potential predictor for severity of obstructive lung disease (79).
Monitoring	NET		Non-type 2	Potential biomarker for monitoring ICS treatment in obstructive lung disease. Patients who have regular ICS show lower levels than those who do not have regular ICS (5).
	Periostin		Type 2	Useful for monitoring anti-IL-13 and anti-IgE treatments (15, 99).
Therapeutic	*IL4R*	rs8832A>G	Type 2	Predict the efficiency of IL-4Rα antagonist drugs (111).
	HSF		Non-type 2	Target for decreasing ECM excess from matrix transfer during lung tissue injury (47).
	IL-35		Non-type 2	Reduces fibrosis expression by preventing TGF-β binding and Th17 differentiation (78).
	Neutrophil integrins		Non-type 2	Novel therapy for non-type-2 inflammation phenotype (49).
	Periostin		Type 2	Target for reducing mucus secretion and eosinophilic inflammation (15).
	Siglec-8		Type 2	Target marker for enhancing apoptosis of eosinophil via ADCC activity and inhibiting mast cell functions (193).
	Eosinophil adhesion		Type 2	Target for reducing survivability and pro-proliferative effects on the lung structure (38).
	IL-3		Type 2	Alternative target for controlling eosinophil activation in anti-IL5 non-responders (39).
	IL-33		Type 2	Target for decreasing eosinophil functions and allergic processes (37).
	Mast cells		Type 2	Mast-cell-depleting monoclonal antibodies decrease disease exacerbation (51).
	PD-1		Type 2	Target for decreasing ILC2 activation and functions (61).
	IL-9		Type 2/Non-type 2	Target for decreasing in Th2 and Th17 cytokine expression, eosinophil infiltration, goblet cell hyperplasia, and the proliferation of ILC2, Th2, Th17, and mast cells (76).

For asthma, eosinophils and type-2 inflammatory cytokines play a vital role in disease pathogenesis. Persistently high type-2 biomarkers are associated with worse asthma outcomes and predict the response to treatment with type-2 biologics such as omalizumab, mepolizumab, benralizumab, and dupilumab (119–121). Conventional systemic biomarkers, including blood eosinophilia, serum periostin, and serum sIgE, have been proven to be predictive markers for the response to omalizumab in landmark studies (122–124). The level of blood eosinophilia has been shown to respond to IL-5 receptor antagonists, such as benralizumab, in a dose-dependent fashion. A significant reduction in asthma exacerbations was noted with increased blood eosinophilia (125, 126).

Exhaled biomarkers such as FeNO are becoming increasingly promising as markers for investigating the upregulation of the IL-4/IL-13 pathway in airway epithelial cells (115, 127). An increase in FeNO, along with higher blood eosinophilia, serves predictive roles in response to IL-4 receptor antagonists or dupilumab for reducing exacerbations (128–130). These findings emphasize the predictive significance of biomarkers related to the immunopathogenesis of the type-2 asthma phenotype.

In contrast to type-2 high asthma, characterized by eosinophilic and allergic inflammation, type-2 low asthma includes not only neutrophilic asthma but also mixed granulocytic and pauci-granulocytic asthma (131, 132). Currently, there is no consensus or clear definition of type-2 low asthma in clinical practice (133). As a result, no neutrophilic biomarkers are available for clinical use (134). Clinical trials have shown that neutrophilic-targeted biologic agents for severe asthma, such as anti-IL-17 and anti-TNF-α antagonists, are ineffective (135, 136). Despite specific antagonists targeting various asthma phenotypes based on inflammatory profiles, an unmet need persists (131). To date, the anti-TSLP monoclonal antibody tezepelumab has shown clinical efficacy in both type-2 high and type-2 low asthma by modulating the upstream cytokine pathways involved in asthma pathogenesis (137–140). However, critical factors for clinical application include cost-effectiveness, availability, accessibility of these treatments, and patient selection (141, 142).

COPD is characterized by corticosteroid insensitivity (143). Both neutrophilic and eosinophilic inflammation underlies the disease pathogenesis (144). The increased blood eosinophilia in COPD is associated with a higher frequency of exacerbations. Therefore, COPD patients with eosinophilia and frequent exacerbations, despite maximizing bronchodilator therapy, are indicators for ICS-containing regimens according to treatment recommendations (145). Nevertheless, the predictive roles of blood eosinophilia for ICS in patients with COPD are widely accepted (146, 147). The role of type-2 inflammation, particularly in COPD, concerning elevated blood eosinophilia has been debated. Recent clinical trials have demonstrated clinical benefits in treating type-2 inflammation with biologics, including IL-4/IL-13, IL-5, and IL-5R modulation. Despite these findings, the clinical trial results for mepolizumab and benralizumab have shown inconsistent outcomes regarding the reduction of exacerbations in COPD patients with blood eosinophilia; however, dupilumab can reduce exacerbations and improve lung function (148–150). Additionally, anti-inflammatory treatments for COPD, including low-dose azithromycin, have been shown to reduce COPD exacerbations (151, 152). However, the precise immunomodulating mechanisms underlying these effects have not been fully elucidated.

Finally, ACO, the distinct clinical entities of pure asthma and COPD have been proposed for two decades (153). The immunopathogenesis of coexisting asthma and COPD has not been fully clarified (154). Since the definitive diagnosis of ACO depends on the presence of both clinical features of asthma and COPD in patients with airway diseases, it remains non-standardized. Consequently, clinical biomarkers for ACO have been insufficient (155–158). More importantly, the primary treatment consists of ICS-containing regimens, which rely on managing asthma and COPD with eosinophilic inflammation as a cornerstone. To date, there is no specific targeted treatment for ACO, and there is a lack of promising biomarkers to predict clinical responsiveness to ACO treatments (159, 160).

The novel molecular and cellular biomarkers that are promising for determining the nature of the disease represent unmet needs and are urgently required for clinical application. The majority of biomarkers used in clinical practice depend on ease of use and convenience in clinical contexts. However, the mechanistic correlation with the pathogenesis of diseases, the correlation with disease severity, and the predictive capability regarding clinical outcomes are lacking (112). More importantly, the majority of clinical trials for targeted therapies for asthma and COPD rely on conventional biomarkers, such as blood eosinophils, serum total IgE, and FeNO (161). Therefore, the off-target approach significantly impacts the clinical efficacy and effectiveness of these treatments. The lack of molecular and target specificity for immunopathogenesis mechanism underlying biologic agents, which modulate both type-2 high and type-2 low phenotypes, remains an unmet clinical need (154).

The present article review focuses on the immunopathogenesis of asthma, COPD, and ACO, emphasizing the role of circulating cytokine assays, cytokine receptor expression on the surface of inflammatory cells, and functional responses in laboratories. Determining whether these factors become targets of type-2 and non-type-2 biological treatment requires further clinical studies. Both the modulation of circulating cytokines and cytokine receptor expression can be measured and applied in future preclinical and clinical studies. Their roles in assessing the efficacy and effectiveness of these targeted treatments for asthma, COPD, and ACO show promise as novel biomarkers (154, 162).

**Figure 4:**
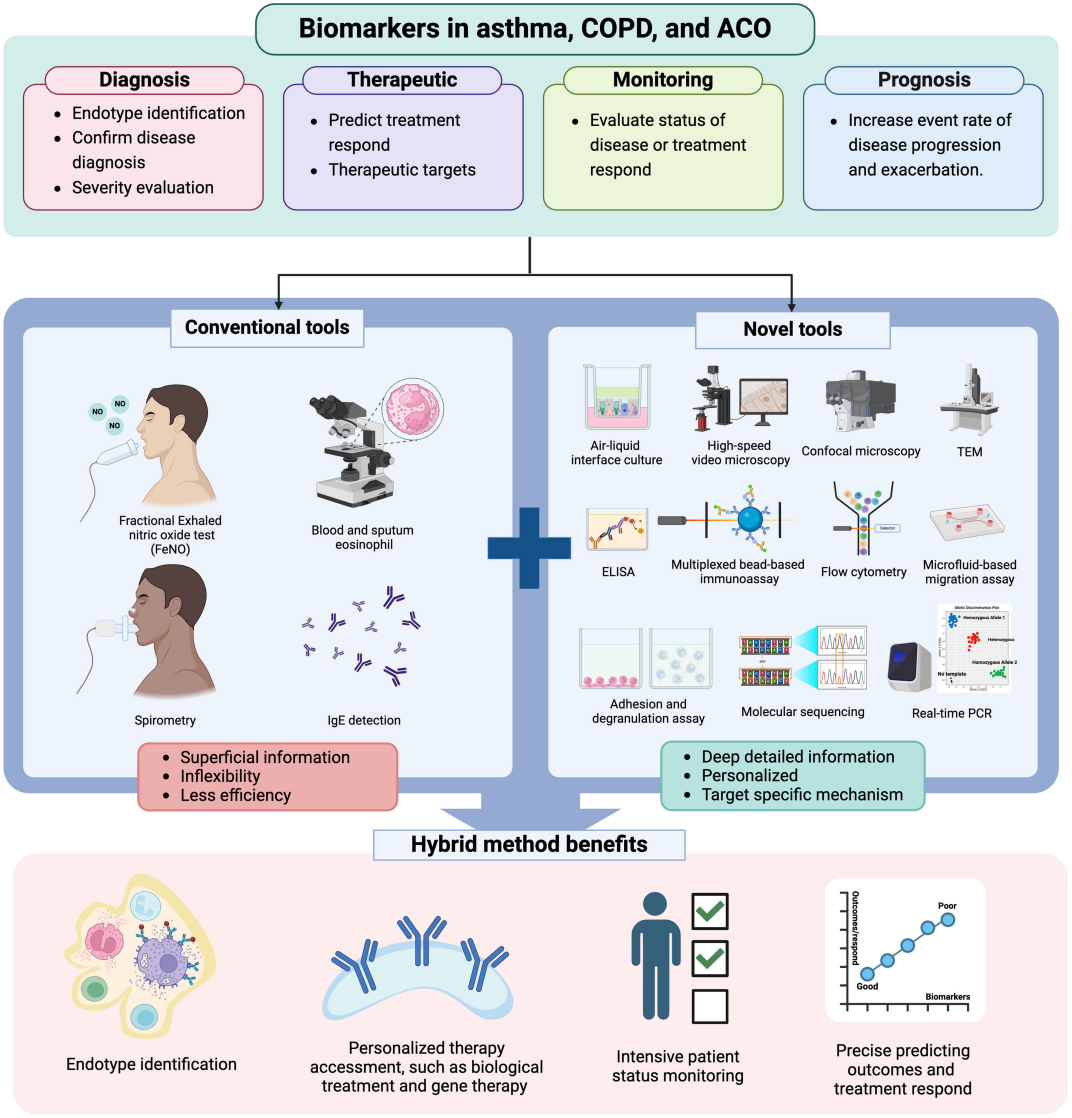
The role of novel tools in future clinical practice. Figure created with www.BioRender.com. Only conventional tools, such as FeNO, blood and sputum eosinophil counts, spirometry, and IgE detection, are insufficient to provide a comprehensive understanding of the patient's actual profile. Therefore, combining novel and conventional tools provides more detailed patient profiles, enabling clinicians to enhance diagnostic accuracy, select the most effective treatments, monitor patient status closely, and predict adverse outcomes or treatment responses. TEM, transmitted electron microscopy; real-time PCR, real-time polymerase chain reaction.

Despite the use of conventional biomarkers in the clinical field to investigate their roles in airway diseases, they do not encompass all patient characteristics (162) (Figure 4). Developing ideal and novel biomarkers is pivotal to improving the outcomes of airway disease management in the future. The goal of these biomarkers is to comprehensively capture individual traits, particularly concerning the immunopathogenesis of obstructive diseases (154). Furthermore, these novel biomarkers may serve as surrogates to guide the development of more treatable targets.

Consequently, the integration of biomarkers into the real-world management of airway diseases, including ACO, informs approaches to patient care and ultimately enhances clinical outcomes. Novel biomarkers should be easily accessible, cost-effective, and minimally invasive, offering more detailed information about an individual's traits for accurate diagnosis, forecasting treatment outcomes, adjusting doses, prognosticating further outcomes, and tailoring personalized treatment (154, 162) (Figures 3, 4).

## Conclusion

In conclusion, using *in vitro* tools highlights that understanding the underlying immunopathogenesis of disease can improve management, paving the way for personalized patient care based on immune characteristics. Further research is essential to better understand the complexity of ACO and identify potential markers for its management in clinical practice.
